# Multimodal Imaging of an Incidental Anomalous Coronary Artery

**DOI:** 10.1016/j.cjco.2022.11.014

**Published:** 2022-11-21

**Authors:** Boyang Liu, Sasha Lalla, Sandeep S. Hothi, Elisa McAlindon

**Affiliations:** a Department of Cardiology, Royal Wolverhampton NHS Trust, Wolverhampton, United Kingdom; b Institute of Cardiovascular Sciences, College of Medical and Dental Sciences, University of Birmingham, Birmingham, United Kingdom

**Anomalous right coronary artery arising from the main pulmonary artery (ARCAPA) is commonly identified on an incidental basis in asymptomatic individuals. This case report of an adult male patient admitted with troponin-positive chest pain highlights the incidental nature via which ARCAPA may present. Invasive coronary angiography suggested a potential anomalous right coronary origin, before computed tomography (CT) coronary angiography confirmed ARCAPA while also identifying extensive, acute bilateral pulmonary emboli, in itself sufficient to explain the presentation. Stress perfusion cardiac magnetic resonance imaging (MRI) accordingly excluded both myocardial infarction and inducible hypoperfusion. We discuss multimodal investigation in the management of incidental ARCAPA.**

A 50-year-old male ex-smoker presented with a 1-day history of central chest tightness and dyspnea. The patient did not report any fever, hemoptysis, or orthopnea. Obstructive sleep apnea was his only comorbidity. He took no medications. Clinical examination was unremarkable with normal jugular venous pressure, heart sounds, and clear lung fields. Clinical observations were as follows: blood pressure 135/82 mmHg, pulse 90 beats/min, oxygen saturation 97% on air, respiratory rate 18 breaths/min.

Electrocardiography (Supplemental Fig. S1) demonstrated a sinus tachycardia at a rate of 120 beats per minute, T-wave inversion in V1 to V3, and minor ST depression in V3. High-sensitivity troponin I rose from 305 to 563 ng/L over the span of 3 hours. Full blood count and inflammatory markers were normal. Polymerase chain reaction analysis for severe acute respiratory syndrome-coronavirus-2 (SARS-CoV-2) was negative. Plain chest radiography demonstrated cardiomegaly, engorged hila, and moderate upper lobe venous blood diversion but no overt pulmonary edema or pleural effusion (Supplemental Fig. S2). Transthoracic echocardiography demonstrated mild concentric left ventricular hypertrophy with good global left ventricular systolic function; only trace tricuspid regurgitation was present. The patient was given a working diagnosis of non-ST-elevation myocardial infarction by the on-call team and was loaded with aspirin and clopidogrel.

## Diagnosis and Management

**Figure 1:**
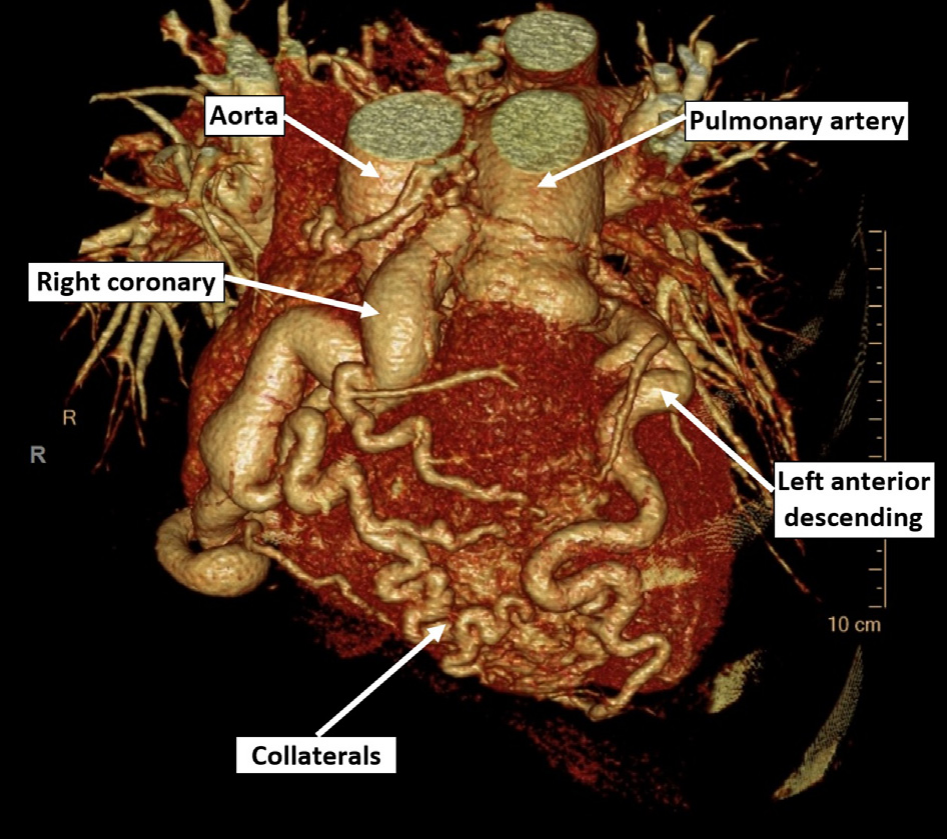
Cardiac CT 3D reconstruction showing dilated LAD and the anomalous dilated and aneurysmal RCA that arises from the pulmonary artery.

On invasive coronary angiography, direct cannulation of the right coronary system failed. Angiography (Video 1 , view video online) of the left coronary artery demonstrates epicardial connection of the right coronary artery to the pulmonary artery. A prospectively gated CT coronary angiogram confirmed the anatomy of the aberrant coronary system with a normal left main stem origin, but with the right coronary artery arising from the main pulmonary artery (ARCAPA; Fig. 1). The coronary system was aneurysmal in all territories, with no focal stenosis, thrombi, or plaques. A retrospective review of the transthoracic echocardiogram highlighted findings that provided the first clue indicating the presence of an ARCAPA, including prominent transeptal collateral vessels on colour Doppler imaging (Video 2 , view video online), pulmonary artery dilatation (pulmonary artery dimension: 34 mm), and a prominent left main coronary artery (Video 3 , view video online). Incidentally, extensive bilateral pulmonary emboli in the main branch pulmonary arteries as well as the segmental arteries were identified as the true cause of troponin elevation (Supplemental Fig. S3), with no contrast reflux into the inferior vena cava.

**Figure 2:**
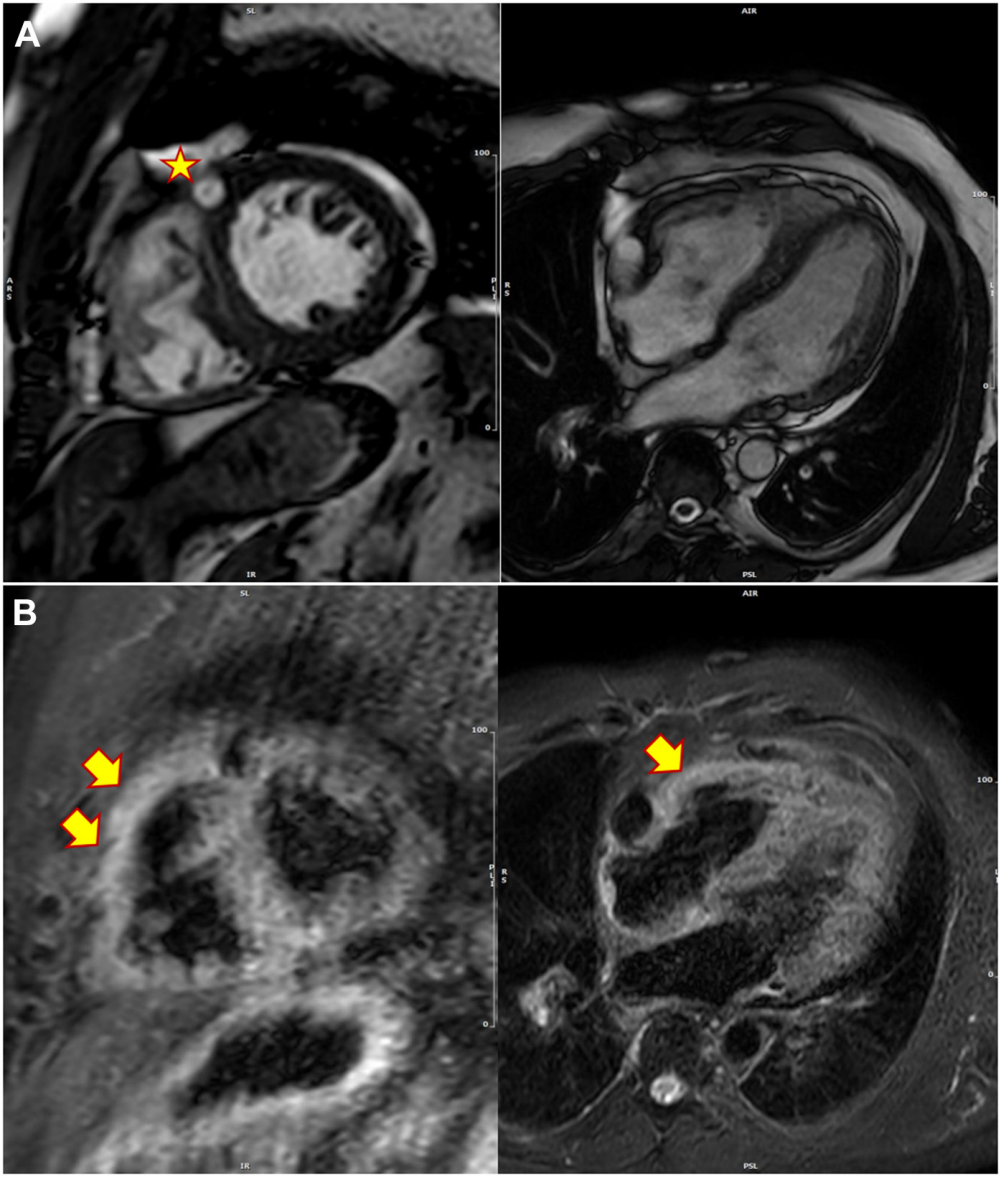
Cardiac MRI cine (**A**) and T2 STIR (**B**) sequences demonstrating the presence of a dilated left anterior descending artery (**star**), with subtle evidence of myocardial oedema in the right ventricular free wall (**arrows**), likely because of pulmonary embolism.

The patient remained pain-free from admission and was commenced on rivaroxaban (15 mg twice daily for 21 days, 20 mg once daily thereafter), and antiplatelet agents were stopped. Inpatient cardiac MRI confirmed an absence of myocardial infarction on late gadolinium enhancement imaging, with good global left ventricular systolic function. Pertinently, right ventricular free-wall edema was identified via T2-weighted short-tau inversion recovery (T2 STIR; Fig. 2), associated with mild right ventricular dilatation according to indexed volume, right ventricular hypertrophy (maximum wall thickness 6 mm), and low-normal right ventricular ejection fraction (52%); these findings correlated with the right precordial T wave inversion and pulmonary embolic findings, consistent with right heart strain.

## Follow-up

The patient remained well throughout admission and was discharged on rivaroxaban. Thrombophilia screening was unremarkable (including CT-thorax-abdomen-pelvis for malignancy; antiphospholipid antibodies, lupus anticoagulant, protein C, protein S, and Factor V Leiden).

Per the European Society of Cardiology (ESC) adult congenital heart disease (ACHD) guidelines,^1^ cases of anomalous origin of the coronary arteries are separated into anomalous left coronary artery from the pulmonary artery and ARCAPA. ALCAPA is associated with a high mortality incidence in infancy; and when diagnosed in adulthood, it is more likely than ARCAPA to be pathologically significant.^2^ Patients may present with symptoms of myocardial ischaemia, ventricular arrhythmia, syncope or heart failure due to left-to-right shunting, and even sudden death.^1^, ^2^, ^3^ The ESC recommends surgery (class I, level of evidence C) in patients with ALCAPA. However, incidental identification of asymptomatic ALCAPA in the elderly is not uncommon,^2^ and as clinicians, we must always adopt a balanced approach to patient care.

Conversely, ARCAPA is commonly a benign condition that is incidentally diagnosed. Surgery for ARCAPA is recommended when symptoms are demonstrably attributable to the coronary anomaly (class I), whereas a class IIa recommendation for surgery is given for those with associated ventricular dysfunction or imaging evidence of ischemia.^1^

During follow-up, our patient underwent a normal outpatient treadmill exercise stress test, reaching heart rate targets after exercising for 10 minutes on the Bruce protocol without ischemic symptoms. Our case was discussed at the regional ACHD multidisciplinary meeting, and the patient has currently opted for a conservative management approach.

## Learning Points

The ESC 2020 ACHD guidelines recommend that all ACHD patients be reviewed once they are in a specialist centre, regardless of the complexity of the heart defect. ARCAPA is a rare condition, with a reported incidence of 0.002%.^4^ Compared with ALCAPA, ARCAPA is more likely to be diagnosed incidentally in older patients, and surgery is indicated only if there is evidence of symptoms, left ventricular dysfunction, or inducible ischemia.

The current case perfectly illustrates the incidental nature of ARCAPA, as well as the unique roles multimodality imaging can play in the evaluation of patients with ACHD. A bedside transthoracic echocardiogram may hint first at a coronary anomaly with features, as in our case. CT coronary angiogram is gold standard for elucidating anomalous coronary anatomy, meanwhile cardiac MRI is superior than transthoracic echocardiography for the structural and functional assessment of the right heart, as well as possessing tissue characterization capabilities. When feasible to perform, exercise stress testing is preferred, as it is deemed to be more representative of normal physiology and has a class IC recommendation for assessing ischemia in patients with anomalous coronary anatomy.^1^ However, direct comparisons between pharmacologic and nonpharmacologic stress are lacking, with limited evidence on the use of pharmacologic stress in children and adults with anomalous coronary anatomy.^5^^,^^6^

**Novel Teaching Points**

• An anomalous coronary artery from the pulmonary artery may be either ALCAPA or ARCAPA.
• ALCAPA is commonly associated with a high incidence of mortality in infants, whereas ARCAPA tends to be incidentally diagnosed in adults.
• Invasive coronary angiography, cardiac MRI, CT, and echocardiography each offers a unique perspective in the evaluation of this condition.
• Surgery for ARCAPA is recommended in the presence of symptoms, ventricular dysfunction, or inducible ischemia.
• Stress testing (exercise, which is preferred over pharmacologic stress) is key in the risk stratification of asymptomatic ARCAPA patients.

## Supplementary Material

Video 1 Right radial approach diagnostic coronary angiogram, right-anterior-oblique cranial projection. Contrast injected into left coronary artery is seen draining via epicardial connections to the right coronary artery into the pulmonary artery.

Video 2 Echocardiography colour Doppler imaging from the apical-4-chamber and parasternal-short-axis views, demonstrating the presence of prominent transeptal collateral vessels that are characteristic for an anomalous origin of a coronary artery arising from the main PA.

Video 3 Transthoracic echocardiogram, parasternal short axis window, demonstrating a dilated main pulmonary artery of diameter 34mm, and a prominent left main coronary artery (arrows).
