# Supplementary material for: Multimodal Imaging of an Incidental Anomalous Coronary Artery
Source: CJC Open. 2022 Nov 21;5(2):161–3. doi: 10.1016/j.cjco.2022.11.014 (PMC9984881; doi:10.1016/j.cjco.2022.11.014)
Supplement: Supplemental Figures S1-S5 [file mmc4.docx]

**Supplementary Figure S1**. Admission ECG demonstrating sinus tachycardia at a rate of 120 bpm, with T wave inversion in leads V1 to V3, and minor ST depression in V3.

**
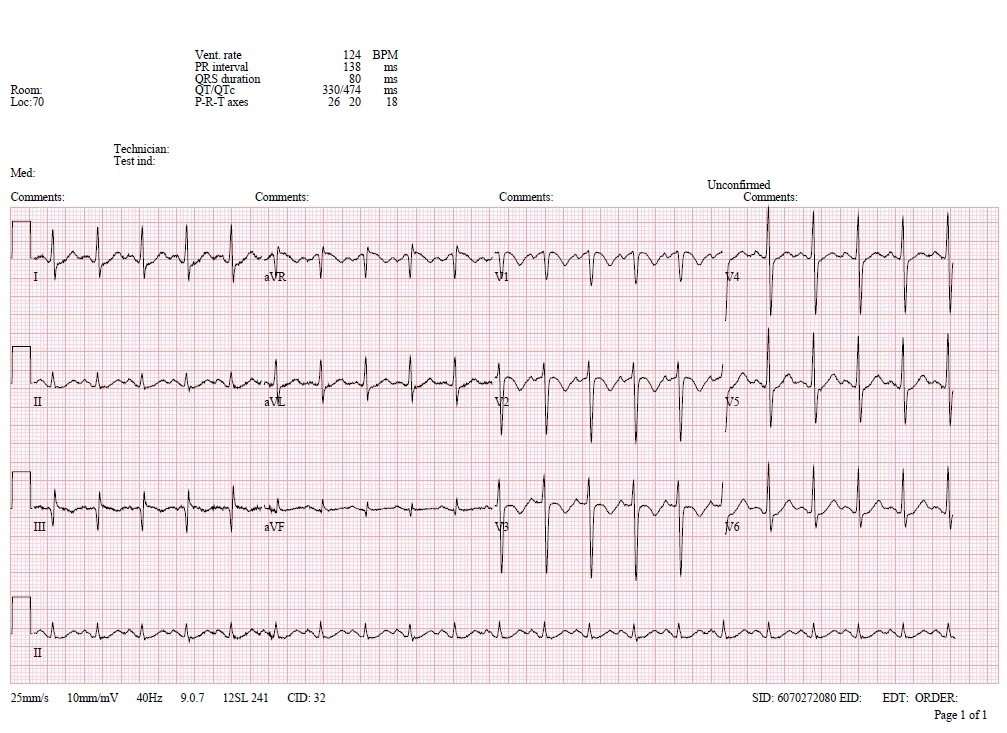
**

**Supplementary Figure S2**. Plain chest radiography demonstrated cardiomegaly with engorged hila and moderate upper lobe venous blood diversion but no overt pulmonary oedema or pleural effusion.

**
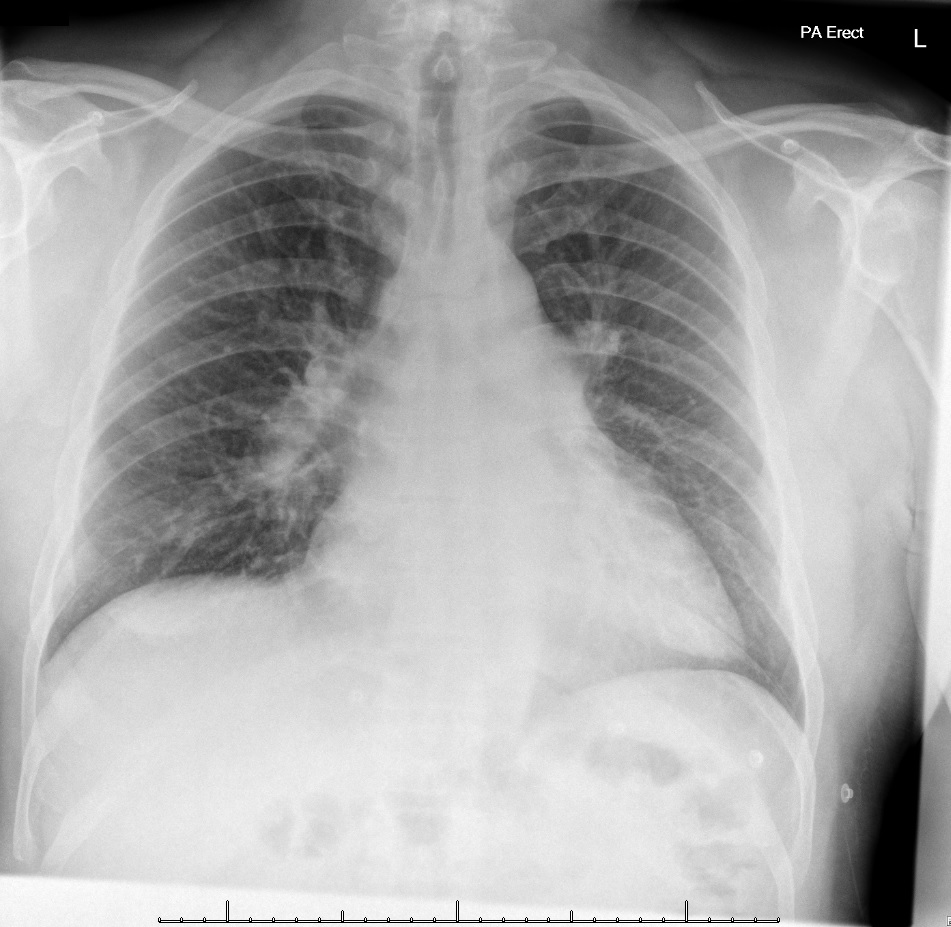
**

**Supplementary Figure S3**. Extensive bilateral pulmonary embolism in both main branch pulmonary arteries on CT angiography.

**
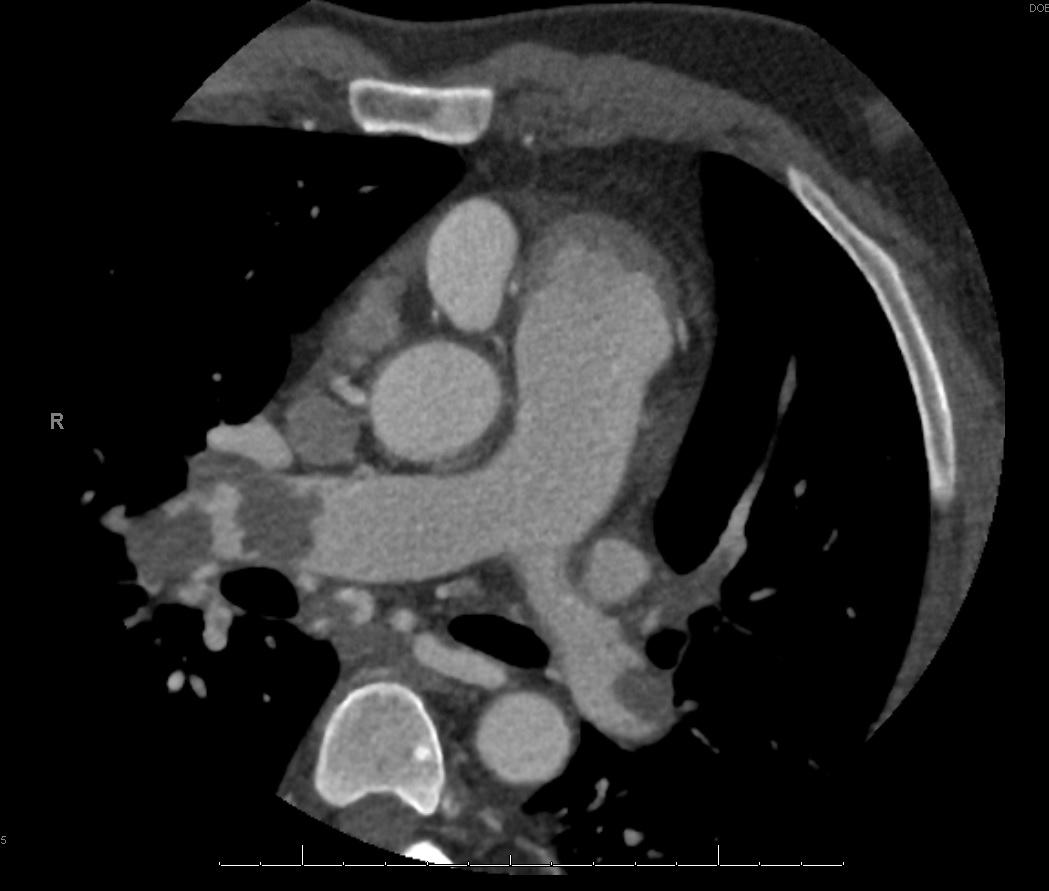
**

**Supplementary Video S1**. Transthoracic echocardiogram, parasternal short axis window, demonstrating a dilated main pulmonary artery of diameter 34mm, and a prominent left main coronary artery (arrows).
